# Two New Cembrane-Based Diterpenoids from the Marine Soft Coral *Sinularia crassa*

**DOI:** 10.3390/molecules17055422

**Published:** 2012-05-08

**Authors:** Yun-Sheng Lin, Nai-Lun Lee, Mei-Chin Lu, Jui-Hsin Su

**Affiliations:** 1 Department of Biological Science and Technology, Meiho University, Pingtung 912, Taiwan; Email: x00010106@meiho.edu.tw; 2 National Museum of Marine Biology &amp; Aquarium, Pingtung 944, Taiwan; Email: love4brat1@yahoo.com.tw; 3 Graduate Institute of Marine Biotechnology, National Dong Hwa University, Pingtung 944, Taiwan; 4 Division of Marine Biotechnology, Asia-Pacific Ocean Research Center, National Sun Yat-sen University, Kaohsiung 804, Taiwan

**Keywords:** diterpenes, soft coral, *Sinularia crassa*

## Abstract

Two new cembrane diterpenes, sicrassarines A and B (compounds **1** and **2**), were isolated from the Taiwanese soft coral *Sinularia crassa*. The structures of the new metabolites were determined on the basis of extensive spectroscopic analysis, particularly mass spectroscopy and 2D NMR (^1^H–^1^H COSY, HMQC, HMBC, and NOESY) spectroscopy.

## 1. Introduction

Marine soft corals of the genus *Sinularia* have attracted a great deal of attention in light of the structural diversity of and wide range of biological activities of their metabolites, including terpenoids and sterols [1]. In previous reports, cembrane-type diterpenes have been stated to occur in relatively few terrestrial organisms, such as tobacco leaves, pine oleoresins and termite secretions. In contrast, they are generally the major components of marine octocorals [2]. Research into the pharmacological properties of this class of natural products is of particular interest. Our previous chemical examination of soft corals of the genus *Sinularia* led to the isolation and identification of various oxygenated cembrane-type metabolites [3,4,5,6,7,8,9]. Some of these have been found to possess several kinds of biological activities, such as cytotoxic [3] and anti-inflammatory properties [4,5,6,8,9]. The current chemical investigation of *Sinularia crassa* led to the discovery of two new cembrane-based diterpenoids, sicrassarines A and B (**1** and **2**). The structures of **1** and **2** were established by detailed spectroscopic analysis, including extensive examination of two-dimensional nuclear magnetic resonance (2D NMR) [1H–1H correlation spectroscopy (COSY), heteronuclear multiple quantum coherence (HMQC) and heteronuclear multiple bond connectivity (HMBC)] correlations. The cytotoxicity of compounds **1** and **2 **against human promyelocytic leukemia (HL60), human breast adenocarcinoma (MDA-MB-231), human colon adenocarcinoma (HCT-116) and human colorectal carcinoma (DLD-1) cell lines was studied.

**Figure 1:**
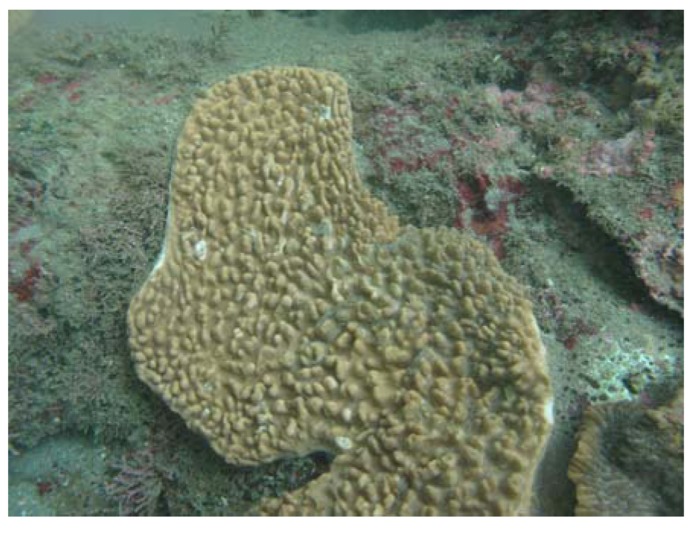
Soft coral *Sinularia crassa*.

## 2. Results and Discussion

**Figure 2:**
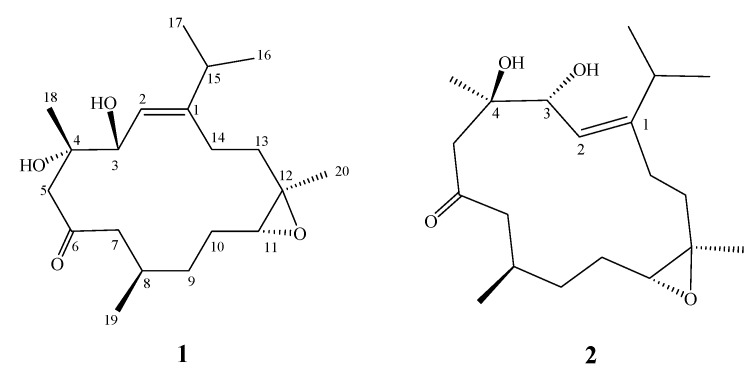
Structures of **1** and **2**.

Frozen samples of *S. crassa* (Figure 1) were extracted with EtOAc. The dry EtOAc extracts were fractionated by silica gel gravity column chromatography, and the eluted fractions were further purified by high pressure liquid chromatography (HPLC) to yield cembranoids **1** and **2** (Figure 2). The new compounds were given the trivial names sicrassarine A (**1**) and sicrassarine B (**2**).

Sicrassarine A (**1**) was isolated as a colorless oil. The high resolution electrospray mass spectra (HR-ESI-MS) spectrum of **1** exhibited a molecular ion peak at *m/z* 361.2358 [M + Na]^+^, with the molecular formula C_20_H_3__4_O_4_, implying four degrees of unsaturation. The infrared (IR) spectrum of **1** revealed the presence of hydroxy (ν_max_ = 3435 cm^−1^) and carbonyl functionalities (ν_max_ = 1700 cm^−^^1^). The ^13^C-NMR data of **1** showed the presence of 20 carbons (Table 1): five methyls, six sp^3^ methylenes, four sp^3^ methines (including two oxygenated carbons at δ 70.8 and 63.7), one sp^2^ methine and four quaternary carbons (including two oxygenated carbons at δ 74.7 and 61.1, one olefinic carbon at δ 152.8, and one keto-carbonyl at δ 212.0). The ^1^H-NMR data (Table 1) revealed the presence of one olefinic proton (δ 5.39, d, *J *= 9.0 Hz) and one oxygenated methine (δ 4.37, d, *J *= 8.5 Hz). A proton signal at δ 2.79 (1H, dd, *J* = 9.5, 4.0 Hz) that correlated with a carbon signal at δ 63.7 in the HMQC spectrum of **1** was attributed to the proton of a trisubstituted epoxide.

**Table 1:** ^1^H and ^13^C NMR datafor **1** and **2**.

	1			2		
	δ_H_ (*J* in Hz) ^a^	δ_C_ (mult.) ^b^		δ_H_ (*J* in Hz) ^a^	δ_C_ (mult.) ^b^	
1		152.8 (C)				152.5 (C)
2	5.39 d (9.0)	119.9 (CH) ^c^		5.07 d (8.0)		120.6 (CH)
3	4.37 d (8.5)	70.8 (CH)		4.62 d (8.0)		71.1 (CH)
4		74.7 (C)				74.4 (C)
5	2.74 d (15.0); 2.67 d (15.0)	51.6 (CH_2_)		2.89 d (18.5); 2.57 d (18.5)		46.2 (CH_2_)
6		212.0 (C)				213.0 (C)
7	2.63 dd (16.0, 9.0); 2.30 m	51.9 (CH_2_)		2.47 dd (14.5, 4.5); 2.05 m		52.8 (CH_2_)
8	2.13 m	29.6 (CH)		1.84 m		31.4 (CH)
9	1.40 m	31.6 (CH_2_)		1.24 m		31.1 (CH_2_)
10	1.91 m; 1.20 m	25.0 (CH_2_)		1.99 m; 1.18 m		26.3 (CH_2_)
11	2.79 dd (9.5, 4.0)	63.7 (CH)		2.65 dd (9.5, 2.5)		62.8 (CH)
12		61.1 (C)				60.0 (C)
13	2.18 m; 1.10 m	37.3 (CH_2_)		2.10 m; 1.74 m		33.1 (CH_2_)
14	2.39 m; 2.16 m	26.0 (CH_2_)		2.21 m; 2.02 m		25.7 (CH_2_)
15	2.28 m	32.0 (CH)		3.08 m		29.7 (CH)
16	1.02 d (7.0)	22.5 (CH_3_)		1.05 d (7.0)		21.7 (CH_3_)
17	1.05 d (7.0)	21.0 (CH_3_)		1.06 d (7.0)		21.2 (CH_3_)
18	1.32 s	24.6 (CH_3_)		1.33 s		22.0 (CH_3_)
19	0.96 d (6.5)	19.9 (CH_3_)		0.98 d (6.5)		20.0 (CH_3_)
20	1.24 s	16.4 (CH_3_)		1.20 s		18.6 (CH_3_)
4-OH				3.86 s		

*^a^* 500 MHz in CDCl_3_; *^b^* 125 MHz in CDCl_3_; *^c^* Numbers of attached protons were deduced by DEPT experiments.

**Figure 3:**
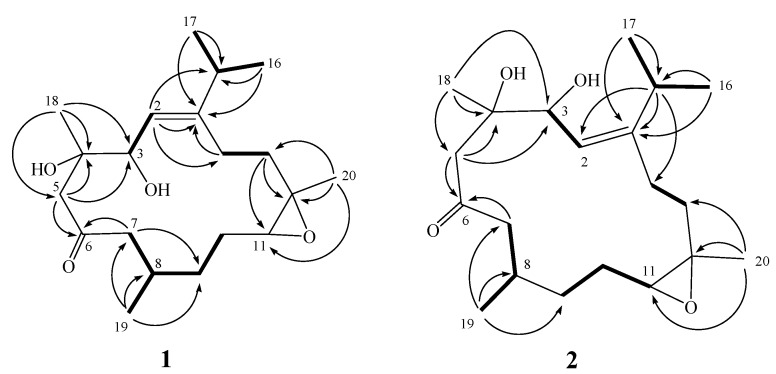
Key ^1^H-^1^H COSY and HMBC correlations of **1** and **2**.

The gross planar structure of **1** was determined by detailed analysis of its 1D and 2D NMR spectra (Figure 3). ^1^H–^1^H COSY spectral analysis established four partial structures of consecutive proton spin systems (Figure 3). These data, together with the HMBC correlations (Figure 3) from H-2 to C-1 and C-14, H_2_-5 to C-3, C-4 and C-6 (carbonyl carbon), H-7 to C-6 and C-9, H_2_-13 to C-11 and C-13, and H_2_-14 to C-1 established the connectivity within the 14-membered ring. The methyl groups attached at C-4, C-8 and C-12 were confirmed by the HMBC correlations from H_3_-18 to C-3, C-4 and C-5, H_3_-19 to C-7, C-8 and C-9, and H_3_-20 to C-11, C-12 and C-13. An isopropyl moiety attached at C-1 was confirmed by the HMBC correlations from both methyl H_3_-16 and H_3_-17 to C-1 and C-15. Thus, **1** was found to possess one trisubstituted olefin at C-1/C-2, one ketone group at C-6, and one trisubstituted epoxide at C-11/C-12.

**Figure 4:**
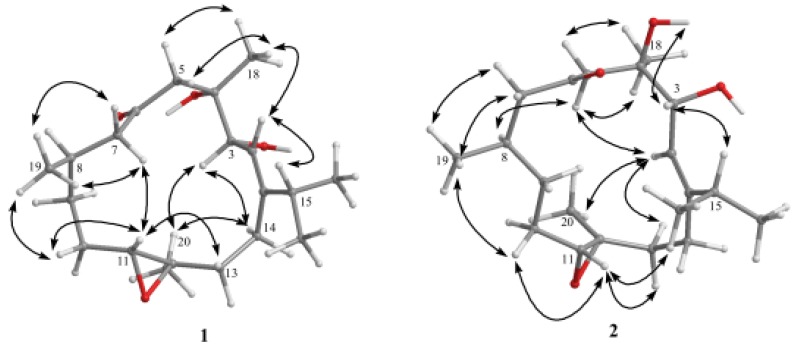
Selective NOESY correlations of **1** and **2**.

The relative configuration of **1**, elucidated mainly from the nuclear Overhauser effect spectroscopy (NOESY) spectrum, was compatible with that of **1** ascertained using molecular mechanics calculations (MM2), which suggested the most stable conformations, as shown in Figure 4. 

In the NOESY spectrum, H-3 showed a nuclear Overhauser effect (NOE) with H_3_-20 but not with H_3_-18. Thus, assuming the *α*-orientation of H-3, H_3_-20 should be positioned on the *α *face. Moreover, H_3_-18 should be positioned on the *β* face. In addition, H-11 was found to interact with one proton of the C-13 methylene (δ 1.10), but not with H_3_-20, revealing the *trans* geometry of the trisubstituted epoxide. In addition, one proton of the C-10 methylene (δ 1.91) was found to exhibit correlations with H-11 and H_3_-19, indicating that these protons are situated on the same face. On the basis of the above findings and other detailed NOE correlations (Figure 4), the structure of **1** was established unambiguously.

Sicrassarine B (**2**) had the same molecular formula (C_20_H_3__4_O_4_) as **1**, as indicated by HR-ESI-MS and NMR spectra (Table 1). Comparison of the ^1^H and ^13^C NMR data of **2** with those of **1** revealed that the two compounds possessed similar structures. The planar structure and all of the ^1^H and ^13^C chemical shifts of **2** were elucidated by 2D NMR spectroscopic analysis, in particular ^1^H–^1^H COSY and HMBC experiments (Figure 3). Thus, **2** was found to possess one trisubstituted olefin at C-1/C-2, two hydroxy groups at C-3 and C-4, one ketone group at C-6 and one trisubstituted epoxide at C-11/C-12. Careful analysis of the NOESY spectrum of **2**, in comparison with that of **1**, allowed determination of the relative stereochemistry of **2**, as shown in Figure 4. Thus, the structure of **2** was established.

Finally, we used a 3-(4,5-dimethylthiazol-2-yl)-2,5-diphenyl tetrazolium bromide (MTT) assay to examine the cytotoxic activities of compounds **1** and **2** against several cancer cells, including HL60 (human promyelocytic leukemia), MDA-MB-231 [human breast adenocarcinoma (grade III)], DLD-1 (human colon adenocarcinoma) and HCT-116 (human colorectal carcinoma) cancer cells. Cells were treated with different concentrations of **1** and **2** for 72 hr. The viability of the various cancer cells was not significantly decreased by 50%, even under treatment with 20 μg/mL of **1** and **2**. In addition, the IC_50 _values of compounds **1** and **2** were both over 20 μg/mL (Table 2). The results showed that these two compounds did not possess cytotoxicity against these cancer cells.

**Table 2:** Cytotoxicity (IC_50_ μg/mL) of compounds **1** and **2**.

	Cell Lines				
Compound	HL60	MDA-MB-231	DLD-1	HCT-116	
**1**	NA *^b^*	NA *^b^*	NA *^b^*	NA *^b^*	
**2**	NA *^b^*	NA *^b^*	NA *^b^*	NA *^b^*	
**Doxorubicin *^a^***	0.058	6.31	5.71	0.51	

*^a^* Clinical anticancer drug used as a positive control. *^b^* NA, not active at 20 μg/mL.

## 3. Experimental

### 3.1. General

Optical rotation values were measured using a Jasco P-1010 digital polarimeter. IR spectra were recorded on a Varian Digilab FTS 1000 Fourier transform infrared spectrophotometer. NMR spectra were recorded on a Varian Unity INOVA 500 Fourier transform-nuclear magnetic resonance (FT-NMR) instrument at 500 MHz for ^1^H-NMR and 125 MHz for ^13^C-NMR, respectively, in CDCl_3_ (Cambridge Isotope Laboratories, Inc.). ESIMS and HRESIMS data were recorded with a Bruker APEX II mass spectrometer. Gravity column chromatography was performed on silica gel (230–400 mesh, Merck). Thin layer chromatography (TLC) was carried out on precoated Kieselgel 60 F254 (0.2 mm, Merck) and spots were visualized by spraying with 10% H_2_SO_4_ solution followed by heating. HPLC was performed using a system comprised of a Hitachi L-7100 pump and a Rheodyne 7725 injection port. A preparative normal phase column (Hibar 250 × 21.2 mm, Supelco, silica gel 60, 5 μm) was used for HPLC.

### 3.2. Animal Material

**Figure 5:**
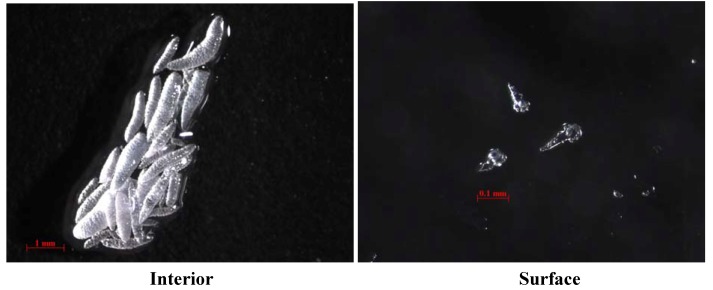
Sclerites of the interior and surface of the colony.

The marine soft coral *S. crassa* (Tixier-Durivault, 1945) was collected by scuba divers at a depth of around 10 m off the coast of Taitung County, Taiwan, in October 2011, and the sample was frozen immediately after collection. A voucher sample was deposited at the National Museum of Marine Biology and Aquarium, Taiwan (specimen no. 2011-1012-7). This species in situ is chip block in shape and appears pale yellow (Figure 1). Under atmospheric conditions, the exterior becomes a medium brown shade while the interior displays a light beige shade. Interior sclerites are large tuberculate spindles and sclerites of surface layer are clubs and small narrow spindles (Figure 5). 

### 3.3. Extraction and Separation

The frozen bodies of soft coral (0.8 kg, fresh wt.) were collected and freeze-dried. The freeze-dried (350 g) material was minced and extracted exhaustively with EtOAc (5 × 1 L). The organic extract was evaporated to yield a residue (10.5 g), which was fractionated by open column chromatography on silica gel using *n*–hexane-EtOAc and EtOAc–acetone mixtures of increasing polarity to yield 15 fractions. Fraction 12, eluting with *n*-hexane-EtOAc (1:1), was further separated by silica gel column chromatography with gradient elution (*n*-hexane-EtOAc, 2:1 to 1:2) to yield five subfractions (12A–12E). Subfraction 12C was subjected to normal phase HPLC with *n*-hexane-acetone (3:1) as the eluent (flow rate 3 mL/min) to obtain compounds **1** (2.5 mg, 0.024% dry wt of extract) and **2** (1.2 mg, 0.011% dry wt of extract). 

Sicrassarine A (**1**): colorless oil; 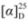 = +77 (*c* 0.2, CHCl_3_); IR (neat) ν_max_ 3435, 2958, 2928, 2870, 1700, 1461 and 1377 cm^−^^1^; ^1^H and ^13^C NMR data, see Table 1; ESIMS *m*/*z* 361 [100, (M + Na)+]; HRESIMS *m*/*z* 361.2358 (calcd. for C_20_H_3__4_O_4_Na, 361.2355).

Sicrassarine B (**2**): colorless oil; 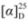 = +37.0 (*c* 0.1, CHCl_3_); IR (neat) ν_max_ 3426, 2957, 2924, 2854, 1697, 1458 and 1375 cm^−^^1^; ^1^H and ^13^C NMR data, see Table 1; ESIMS *m*/*z* 361 [100, (M + Na)+]; HRESIMS *m*/*z* 361.2358 (calcd. for C_20_H_3__4_O_4_Na, 361.2355).

### 3.4. Cytotoxicity Testing

Cell lines were purchased from the American Type Culture Collection (ATCC). Cytotoxicity assays of compounds **1** and **2** were performed using the MTT [3-(4,5-dimethylthiazol-2-yl)-2,5-diphenyl tetrazolium bromide] colorimetric method [10,11]. 

### 3.5. Molecular Mechanics Calculations

Implementation of the MM2 force filed in Chem3D Pro software [12] was used to calculate the molecular models.

## 4. Conclusions

In previous studies, a series of novel secondary metabolites, including sphingosines [13,14], steroids [15] and cembranoids [16], have been isolated from the soft coral *Sinularia crassa*. Among these compounds, one sphingosine and two cembranoids have been found to possess anti-inflammatory activity [13,14,16]. Furthermore, one steroid has been found to possess 5α-reductase inhibitory activity [15]. Our ongoing investigation on the chemical constituents of soft coral *S. crassa* has now led to the isolation and identiofication of two new cembranoids. The present investigation demonstrated that the two metabolites **1** and **2** were inactive (IC_50_’s > 20 μg/mL) towards the growth of HL60, MDA-MB-231, DLD-1 and HCT-116 cancer cells. 

## Supplementary Materials

Supplementary materials can be accessed at: http://www.mdpi.com/1420-3049/17/5/5422/s1.
