# Supplementary material for: Two New Cembrane-Based Diterpenoids from the Marine Soft Coral Sinularia crassa
Source: Molecules. 2012 May 8;17(5):5422–9. doi: 10.3390/molecules17055422 (PMC6268420; doi:10.3390/molecules17055422)
Supplement: Supplementary file 1 [file molecules-17-05422-s001.pdf]

Supporting Information

Two New Cembrane-Based Diterpenoids from the Marine Soft Coral *Sinularia crassa*

Yun-Sheng Lin, Nai-Lun Lee, Mei-Chin Lu* and Jui-Hsin Su*

Table of Contents

S1. Table of Contents

S2. ^{1}H NMR spectrum of **1** in $CDCl_3$ at 500 MHz.

S3. ^{13}C NMR spectrum of **1** in $CDCl_3$ at 125 MHz.

S4. ^{1}H NMR spectrum of **2** in $CDCl_3$ at 500 MHz.

S5. ^{13}C NMR spectrum of **2** in $CDCl_3$ at 125 MHz.

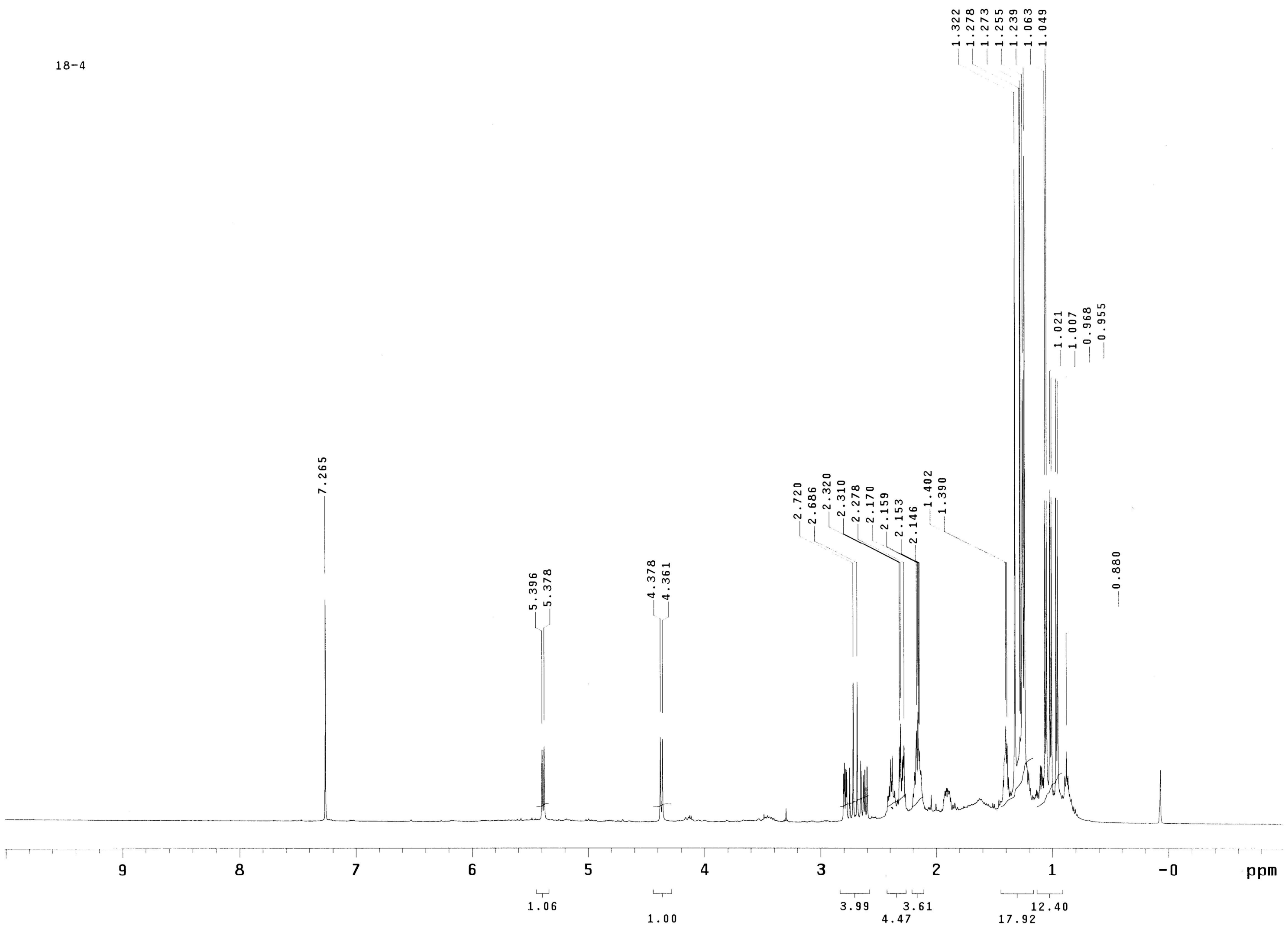

S2. ^{1}H NMR spectrum of **1** in $CDCl_3$ at 500 MHz.

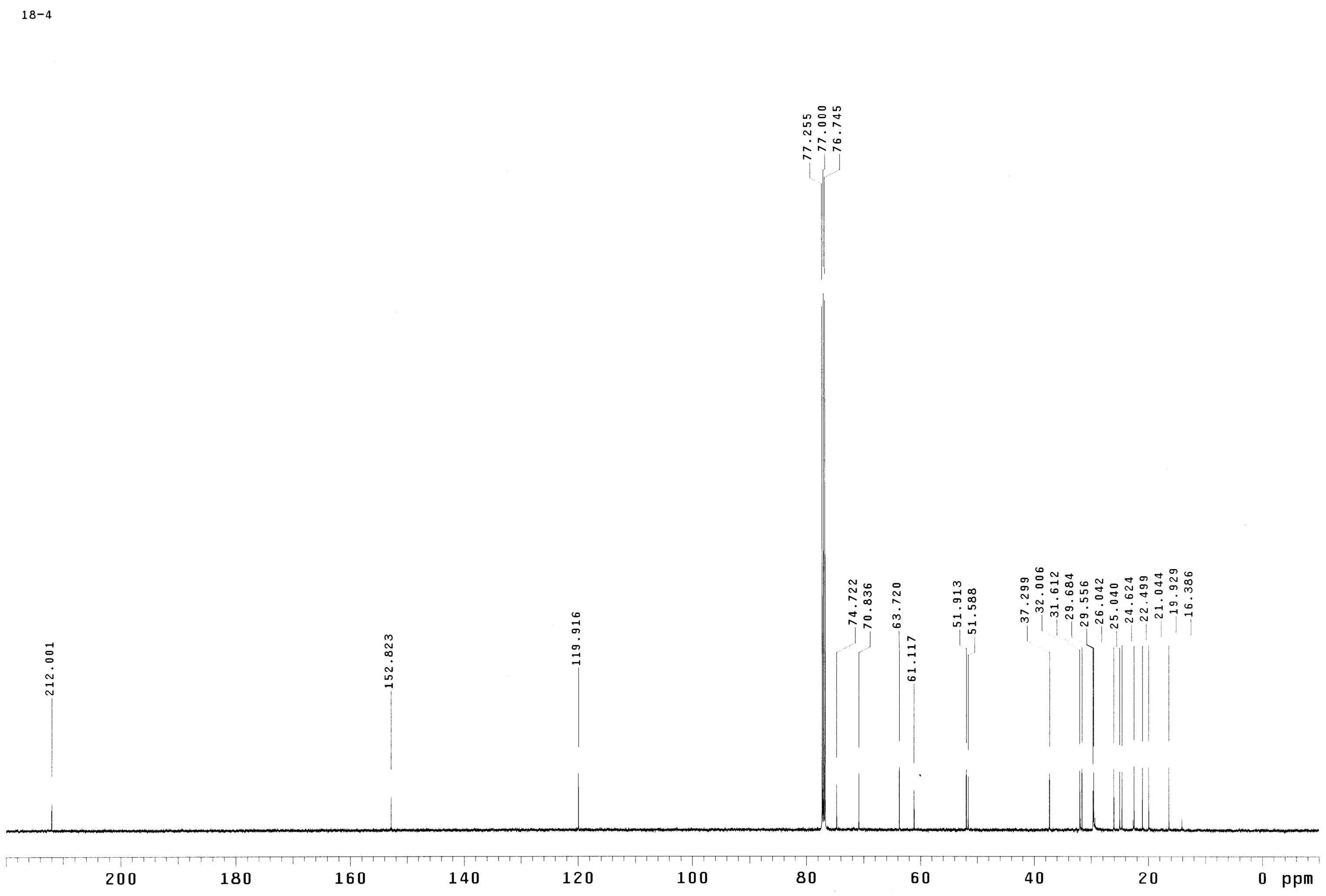

S3. ^{13}C NMR spectrum of **1** in $CDCl_3$ at 125 MHz.

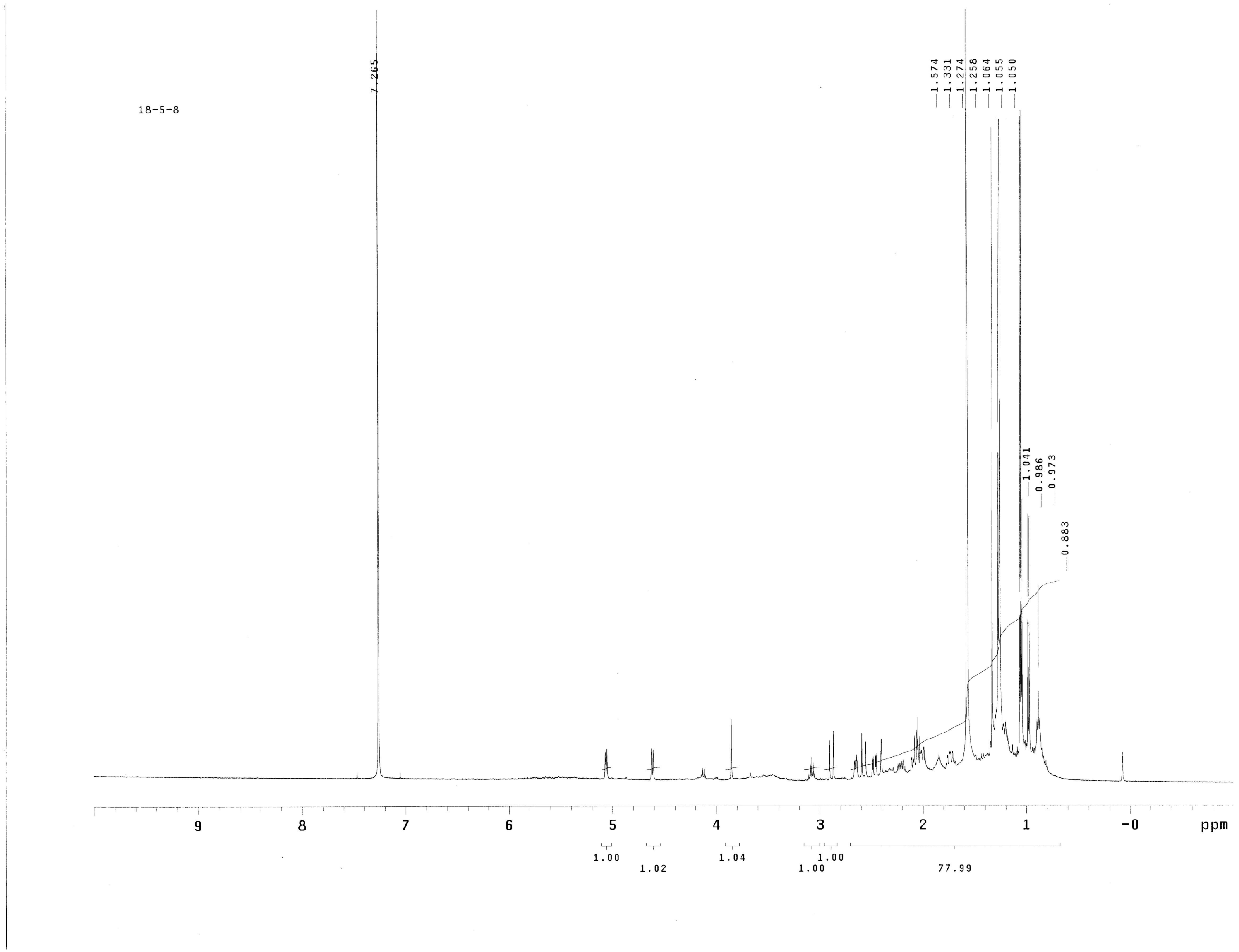

S4. ^{1}H NMR spectrum of **2** in $CDCl_3$ at 500 MHz.

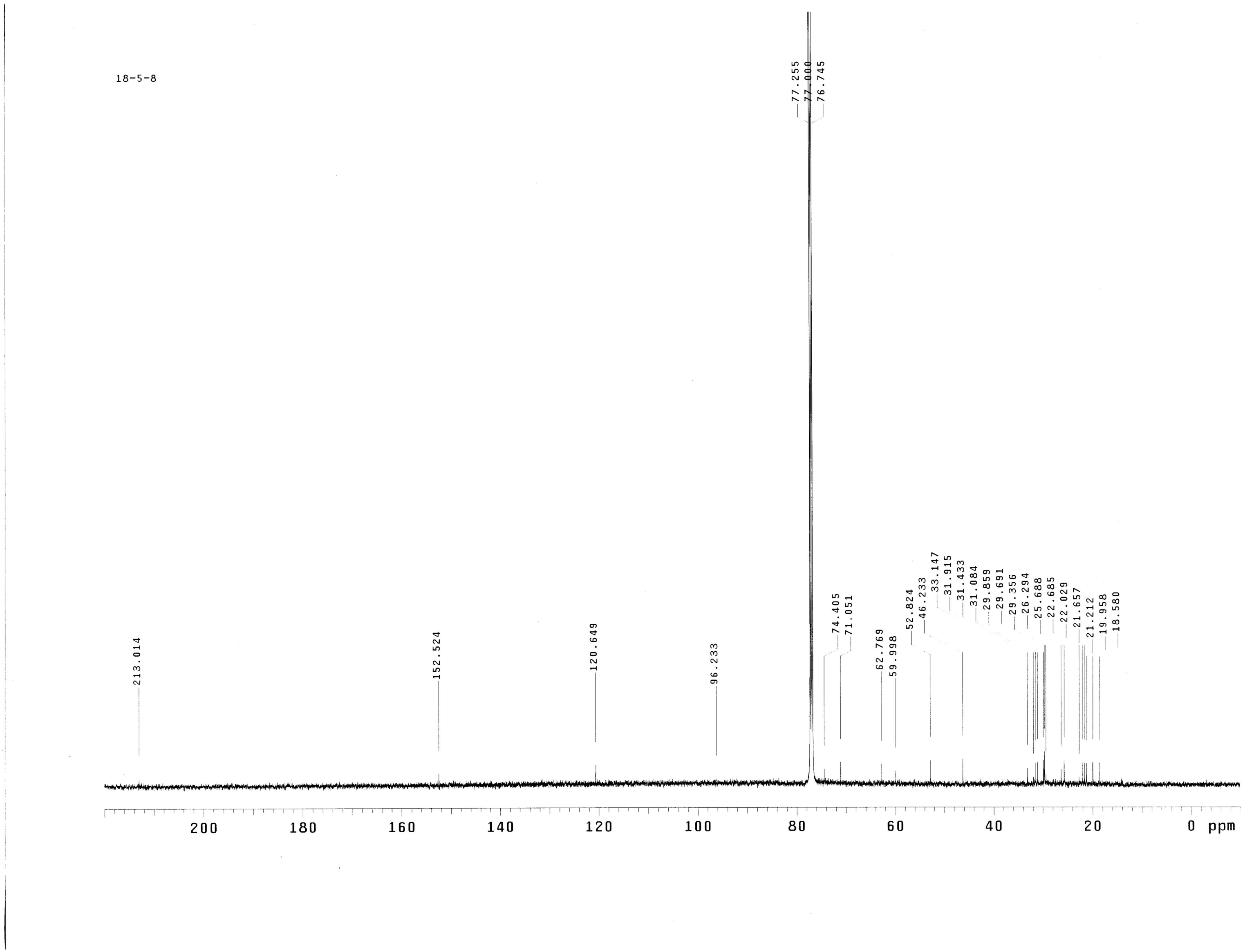

S5. ^{13}C NMR spectrum of **2** in $CDCl_3$ at 125 MHz.
